# Account of Diseases in an Eastern District of London

**Published:** 1802-06

**Authors:** 


### ACCOUNT OF DISEASES IN AN EASTERN DISTRICT OF LONDON,

*From April* 20, *to May* 20, 1802.

| ACUTE DISEASES. | |
|---|---|
| Typhus | 3 |
| Catarrh | 12 |
| Pneumonia | 7 |
| Rheumatifmus Acutus | 5 |
| **CHRONIC DISEASES.** | |
| Tuffis | 17 |
| Dyfpnœa | 18 |
| Tuffis cum Dyfpnœa | 25 |
| Phthifis Pulmonalis | 5 |
| Raucedo | 3 |
| Hydrothorax | 2 |
| Afcites | 2 |
| Anafarca | 4 |
| Cephalalgia | 6 |
| Apoplexia | 1 |
| Paralyfis | 3 |
| Epilepfia | 1 |
| Syncope | 2 |
| Gaftrodynia | 5 |
| Enterodynia | 4 |
| Hæmorrhois | 6 |
| Dyfuria | 4 |
| Calculus | 1 |
| Scrophula | 7 |
| Itch | 3 |
| Herpes | 6 |
| Rheumatifmus Chronicus | 20 |
| **PUERPERAL DISEASES.** | |
| Menorrhagia Lochialis | 3 |
| Maftodynia | 4 |
| Enurefis | 2 |
| **INFANTILE DISEASES.** | |
| Pertuffis | 9 |
| Rubeola | 7 |
| Scrophula | 5 |
| Herpes | 3 |
| Tabes | 2 |

The degree of cold which has prevailed during the period that has paffed fince our laft Report, has been uncommonly fevere; it is reported that, in fome parts of the country, it has been greater than can be recollected to have exifted at the fame feafon of the year. The effects of this have been feverely felt amongft the vegetable tribes; many of the early crops appearing in our gardens a few weeks ago, and which were in a very healthy and flourifhing ftate, are entirely deftroyed. Its effects have alfo been felt in the human frame. Many of the difeafes which commonly retire at this feafon of the year, have either been protracted beyond the ufual period, or have returned with increafed violence. A large number of cafes of catarrh, dyfpnœa, cough, and rheumatifm have occurred. The pretty conftant appearance of fun-fhine, and the pleafant temperature which was felt when fcreened from the north and north-eaft winds, encouraged invalids to venture abroad without fufficient clothing to defend them from the influence of thefe winds; and the confequence has been, a renewal of thofe complaints from which they were gradually recovering.

The difeafes of children ftill prevail; meafles and hooping cough continue to form a large proportion of the diforders to which this clafs of patients is liable. In fome cafes, thofe complaints, or their confequences, have probably been aggravated vated by a removal to different fituations, with a view to change the air, in which they have been more expofed to the cold winds which have been blowing for fome time, and the effects of which they have felt in a feverer degree.

